# Orientation of Head Lice on Human Hosts, and Consequences for Transmission of Pediculosis: The Head Lice Movement Studies

**DOI:** 10.3390/tropicalmed2020011

**Published:** 2017-05-22

**Authors:** Jorg Heukelbach, André Asenov, Fabíola Araújo Oliveira, Iana Lícia Araújo de Melo, Jéssica dos Santos Queiroz, Rick Speare, Uade Samuel Ugbomoiko

**Affiliations:** 1 Department of Community Health, School of Medicine, Federal University of Ceará, Fortaleza CE 60430-140, Brazil; fraubiola@gmail.com (F.A.O.); Iana_mel@yahoo.com.br (I.L.A.d.M.); jessica.squeiroz@yahoo.com.br (J.d.S.Q.); 2 College of Public Health, Medical and Veterinary Sciences, Division of Tropical Health and Medicine, James Cook University, Townsville, Queensland 4811, Australia; 3 Department of Hygiene and Microbiology, Charité Medical School, Campus Benjamin Franklin, D-10117 Berlin, Germany; andreasenov@hotmail.com; 4 Parasitology unit, Department of Zoology University of Ilorin, Kwara State, Nigeria; samugbomoiko@yahoo.com

**Keywords:** *Pediculus humanus capitis*, head lice biology, entomology

## Abstract

We performed head lice movement studies to elucidate factors influencing orientation and movement of head lice. Studies included observation of lice movements on hand and forearm at different positions of the upper limb; movements exposed to unshaved and shaved forearm; and movements with and without antennae. In 57 of 60 (95.0%) observations while holding the hand down, lice moved proximal, and 3 (5%) distal. While holding the hand up, 37/60 (61.7%) moved proximal, and 23 (38.3%) distal (*p* < 0.0001). On the unshaved limb, 29/30 (96.7%) moved proximal, with clockwise movements in 26/30 (86.7%). After shaving, 9/30 (30%) walked proximal and 18 (60%) distal, with 12/30 (40%) clockwise movements. After antennectomy, while holding the hand up, 16/25 (64%) lice did not move, 1 (4%) walked proximal, and 8 (32%) distal. While handing the hand down, 17/25 (68%) did not move, 5 (20%) walked proximal, and 3 (12%) distal. Transmission of head lice may not only occur by head-to-head contact, but also via head-to-body contact, with movement to the head against gravitational pull. Surface factors of hand and forearm may be important in orientation for lice, in addition to gravity. Movement of lice against gravity is not governed by organs in the antennae.

## 1. Introduction

Although infestation with the head louse *Pediculus humanus capitis* (Anoplura: Pediculidae) is highly prevalent in many societies, and pediculosis has been studied scientifically for centuries, similar to other ectoparasites, much is still unknown about their biology [1]. Many of the basic questions about biology were answered in a landmark study performed in 1917 [2]. In that study, Nuttall (1917) reported on the biology of head lice, describing behavior, movement, factors that influenced movement (orientation), and a series of experiments about transmission. He described direct (host-to-host) and indirect (fomite-to-head) transmission modes. Behavior like hiding from light, going upwards or horizontal faster than downwards, and clinging and walking skills on hair were described. He and other authors of that time reported that head lice may be found on the body when numbers of lice were high, but nowadays this finding is extremely rare [2,3,4,5]. Howell (1917) noted that *P. humanus capitis* had a distinct tendency to migrate to the head, which was decreased after several generations of lice were reared in artificial chambers attached to the body [3]. Infestations at former times—when these historical reports were written—differed from the typical infestations in current societies, with mainly schoolchildren and their contact persons being affected.

However, there is still very little evidence on what factors influence movement of head lice. Understanding these would be helpful to further elucidate transmission routes and spread of the ectoparasite in modern societies, and to provide further evidence for the design of effective control measures. We had observed that head lice accidentally displaced onto clothes of people while screening children for pediculosis, the lice invariably started to climb towards the head. In pilot studies, we also observed that lice, when placed on the hands of investigators and left untouched, were detected on the head of these investigators a few days later, suggesting that lice may use gravity for orientation. Thus, we performed a series of studies to elucidate factors that may influence movement of head lice, other than reaction to light (heliotropism) and temperature (thermotropism), as described in the historical literature.

## 2. Materials and Methods

### 2.1. Head Lice

Adult male and female head lice (*Pediculus humanus capitis*) were obtained from infested individuals living in a slum in the city of Fortaleza (northeast Brazil) by dry combing with a fine-toothed plastic comb of good quality. In the community, head lice were hyperendemic [6]. Lice were obtained from individuals that had not treated their infestations with topical or oral antiparasitic compounds, or taken oral antibiotics (as these may kill symbiotic bacteria in lice and subsequently kill them), in the previous four weeks. Before testing, lice were blood-fed on the dorsum of the hands of investigators to maximize activity and movements during testing.

### 2.2. Study Design

Four related studies were performed using human subjects to evaluate the importance of gravity, anatomical surface, and the function of antennae for orientation. Table 1 gives an overview of the studies performed and methods used. All studies were performed during a period of four weeks.

#### 2.2.1. Head Lice Movement Study I

**Figure 1:**
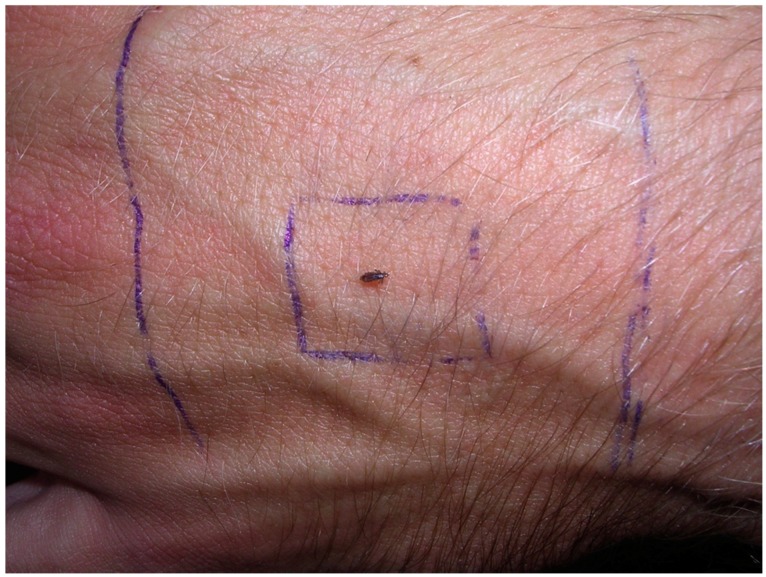
Head lice movement study I. Hair growth direction on the left hand counter-clockwise, on the right hand clockwise.

Fully active adult lice were placed at the center of a 1 × 1 cm square drawn on the middle of the back of the hand of a male investigator with two lines in parallel 2 cm proximal and distal to the edges of the square (Figure 1). General direction of movement (distal/proximal) and type of movement (clockwise/counterclockwise/not circular) were noted when each louse passed a line. For both hands, two positions of the upper limb were used: vertical with hand-highest or vertical with hand-lowest. Lice were observed for up to 10 min; when a louse passed a line, it was removed. Thirty individual lice were used for each hand and position, giving a total of 120 lice. The direction of hair growth on the left hand was counterclockwise and on the right hand clockwise.

#### 2.2.2. Head Lice Movement Study II

**Figure 2:**
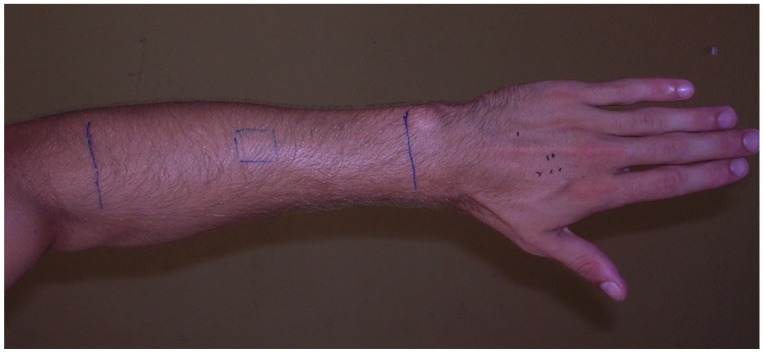
Head lice movement study II. Movement of head lice on forearm, against direction of hair growth (clockwise).

To further investigate how head lice orientate according to the influence of gravity, the procedures of Study I were modified: fully active adult lice were placed on the dorsal aspect of the forearm in a square of 1 × 1 cm, and a line was drawn 10 cm distal and 10 cm proximal to the edges of the square to enable assessment of distance moved (Figure 2). Additional to Study I positions (vertical with holding hand up or vertical with holding hand down), a third position, upper limb horizontal, was also used. Evaluations were performed after 5, 10, 20, and 30 min. For each of the three positions, 30 head lice in batches of 10 were observed; a total of 90 lice.

#### 2.2.3. Head Lice Movement Study III

**Figure 3:**
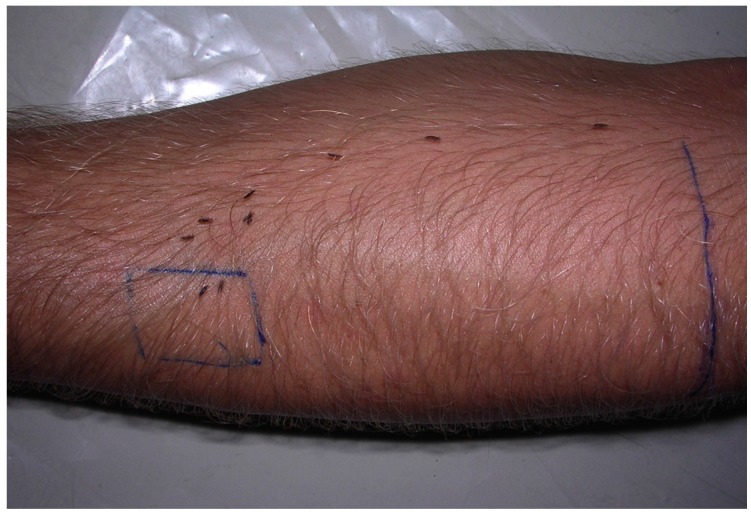
Head lice movement study III. Movement of head lice on unshaved forearm, against direction of hair growth (clockwise).

To identify the importance of the direction of hair growth for orientation and movement of head lice, we performed Study III. We observed walking direction and type of movement of lice on the forearm of a male investigator (Figure 3). Firstly, a baseline was established using the forearm held horizontal. Secondly, the hair of this forearm was shaved, and the study repeated. For each situation, unshaved and unshaved forearm, 30 individual lice were used, making a total of 60 lice. Observation time was 10 min for each phase.

#### 2.2.4. Head Lice Movement Study IV

**Figure 4:**
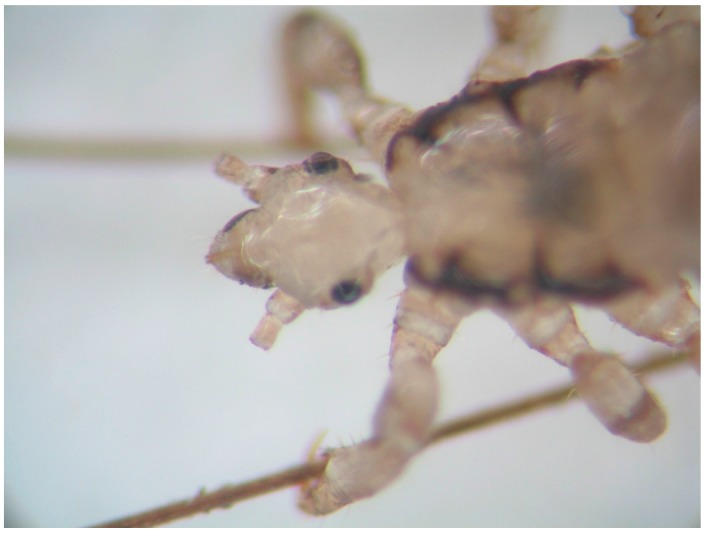
Head lice movement study IV—head louse after amputation of the distal antennae.

We performed Study IV to observe orientation of head lice with and without antennae. The distal parts of both antennae of 43 adult lice were amputated with a scalpel blade under a dissecting microscope, by cutting against a hard surface (Figure 4). Only 25 lice survived this procedure and these were used to study direction of movement for three positions of the upper limb: horizontal, vertical hand-highest, and vertical hand-lowest. Lice were positioned individually on the center of the forearm of a female investigator and tested for all three upper limb positions. Direction of movement of lice was observed. The control group of 25 lice had intact antennae.

### 2.3. Statistical Analysis

Data were entered using Excel spreadsheets and checked for entry errors. Binomial 95% confidence intervals were calculated using STATA, version 10 (Stata Corporation, College Station, TX, USA). Chi squared test was used to compare relative frequencies.

### 2.4. Ethics

The study was approved by the Ethical Review Board of the Federal University of Ceará, Fortaleza, Brazil. Informed written consent was obtained from individuals or guardians of children before combing. After combing, individuals and their families were treated against head lice with oral ivermectin, or in the case of contra-indication, with a permethrin-based over-the-counter product. In Brazil, oral ivermectin is registered for treatment for pediculosis, scabies, and intestinal helminths and has been used effectively for community treatment against pediculosis and other parasites [7,8].

The studies have been presented at the Fourth International Congress on Phthiraptera (ICP4) in Cappadocia/Turkey in 2010.

## 3. Results

### 3.1. Study I

All lice passed a line. While holding the hand down, 95% of lice moved proximal (Table 2). In contrast, while holding the hand up, 62% moved proximal (*p* < 0.0001). Eighty percent (96/120) moved against the direction of hair growth.

### 3.2. Study II

**Figure 5:**
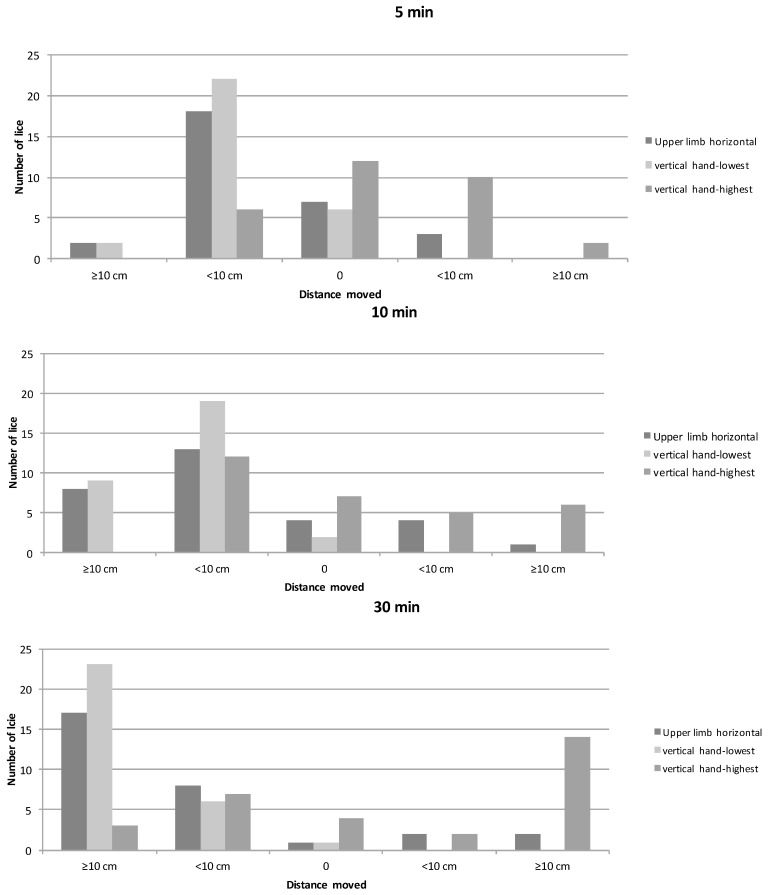
Results of head lice movement study II. Movement of head lice under influence of gravitational pull after 5, 10, 20, and 30 min observation time. The *X*-axis indicates distance moved with proximal to left of zero and distal to right of zero.

In the horizontal position, 83.3% (25/30) of lice moved proximal after 30 min, with 56.7% moving >10 cm and 26.7% <10 cm (Figure 5). Four (13.3%) lice moved distal and only one (3.3%) louse did not change position. While holding the hand down, 96.7% (29/30) lice moved proximal, with 76.7% >10 cm and 20% <10 cm. No lice moved distal, and one (3.3%) louse did not change position. While holding the hand up, 33.3% moved proximal, with 10% moving >10 cm and 23.3% <10 cm; 53.3% moved distal, and 13.3% did not move distal nor proximal. In this position, after 10 min 40% had moved proximal.

### 3.3. Study III

On the unshaved and shaved forearm, 97% and 30% of lice moved proximal respectively. On the unshaved forearm, 87% walked against the direction of hair growth (clockwise). On the shaved forearm, 40% moved clockwise with 47% not showing any circular movements (Table 3).

### 3.4. Study IV

For all three positions, a higher proportion of lice with amputated distal antennae did not move, and this was constant at about 65% (Table 4). When these lice were removed from calculations, both groups of lice showed the same general movement directions; for intact and operated lice respectively: with hand up 82% (14/17), and 89% (8/9) distal movement; with hand down 100% (15/15) and 63% (5/8) proximal movement; and horizontal 100% (16/16) and 88% (7/8) proximal movement.

## 4. Discussion

We present the first systematic study on head lice movement on the human host for many years. The results show that head lice move against the gravitational pull and apparently can detect gravity. Gravity may be one of several factors important for orientation of head lice. The studies also indicated that surface anatomical factors of the hand and forearm, such as the presence of hair and its direction of growth, may be important in orientation. Although we have also shown that amputation of the distal antennae changed the proportion of head lice that moved, the mobile lice followed the trends in the control group in roughly equivalent proportions. This indicates that the physical impact of antennectomy is most likely the explanation for the reduction in mobile lice rather than the antennae housing gravity sensing organs per se. Each antennae has an elaborate set of sensory organs; sharp and blunt peg organs at the tip, tuft organs and pore organs. These are located on the fifth and the fourth segments of the antennae, which were surgically removed in Study IV. As known from studies on body lice (*Pediculus humanus corporis*) the peg organs are associated with olfaction and the tuft organs with detection of relative humidity and possibly temperature [9]. The function of the pore organs is unknown, but they may also have an olfactory function [10].

In spite of the likely damage associated with surgery, this study suggests that the tendency of lice to move against gravity is not governed by sense organs in the antennae. 

It seems that head-to-head transmission is most important for head lice to spread to another host. Head lice use specific cues to transfer, the highest probability being to a moving hair approaching above the louse at 90 degrees to its long axis [11]. Transmission of head lice is still a matter of vigorous discussion, principally the importance of fomite transmission versus head-to-head transmission [11,12,13,14]. While in vitro studies have shown that fomite transmission is possible, field evidence indicates that the primary mechanism is by head-to-head transmission [15]. In fact, some studies have shown that fomites such as hats, pillow cases, brushes, and combs are unlikely to play an important role [16,17].

Our data suggest that, in addition to head-to-head and fomite transmission, head-to-body transmission may occur in some circumstances, with consequent movement of lice from any body part to the head of the new host. There are several possible ways how lice may be transferred from one host to another, without being by fomite transmission nor direct head-to-head contact. Staff of head lice clinics may be infested during combing and also during application of hot air treatments that may blow off viable lice from the hair. In fact, head-to-body transmission has been observed by RS during head lice diagnosis when a louse is expelled from dry hair being combed vigorously by a plastic comb prior to use of a nit comb. Some lice can be suddenly ejected from the hair onto the clothes of the person doing the combing. Apparently this is due to lice and hair carrying the same static electric charge and the louse being repelled. We have then seen these lice begin walking upwards. This event may explain why the prevalence of pediculosis in a classroom-based trial was higher in the group that combed their hair than in the classes that did not [18]. Other factors that were not assessed in our studies, such as hair density and length, and superficial skin factors which may also play a role and should be subject of future studies.

Lice dislodged to any body part of another host may use gravity and direction of hair growth to crawl towards the hair of the head. Although this route of transmission is unlikely to be of major importance on the community level, it does add to the knowledge about this fascinating beast and highlights that care is needed in specific situations.

## Figures and Tables

**Table 1:** Overview of head lice movement studies performed.

	Objective	Approach
Studies I + II	Identify importance of gravity and body hair growth as mechanisms of orientation	Lice placed on hand (study I) or forearm (study II)—observation of movements at different positions of upper limb.
Study III	Identify importance of body hair for movement and orientation	Observation of walking direction and type of movement of lice exposed to unshaved and shaved forearm.
Study IV	Describe importance of antennae for orientation	Observation of direction of movement with and without antennae, for different positions of the upper limb.

**Table 2:** Head lice movement study I—importance of gravity on direction of lice movement, and type of movement.

	N (%)
**Direction of Movement: Holding Hand Down (*n* = 60)**	
proximal	57 (95%)
distal	3 (5%)
**Direction of Movement: Holding Hand Up (*n* = 60)**	
proximal	37 (61.7%)
distal	23 (38.3%)
**Circular Movement: Holding Hand Down (*n* = 60)**	
against hair growth	51 (85%)
according to hair growth	-
no circular movement	9 (15%)
**Circular Movement: Holding Hand Up (*n* = 60)**	
against hair growth	45 (75%)
according to hair growth	4 (6.7%)
no circular movement	11 (18.3%)

**Table 3:** Head lice movement study III—walking direction and type of movement of lice on unshaved and shaved horizontal forearm.

	Unshaved Forearm (*n* = 30)	Shaved Forearm (*n* = 30)
**Direction of Movement**		
proximal	29 (96.7%)	9 (30%)
distal	1 (3.3%)	18 (60%)
no directed movement	-	1 (3.3%)
walked lateral and fell off the limb	-	2 (6.7%)
**Circular Movements**		
clockwise (against direction of hair growth)	26 (86.7%)	12 (40%)
counter-clockwise (with direction of hair growth)	3 (10%)	4 (13.3%)
no circular movements	1 (3.3%)	14 (46.7%)

**Table 4:** Head lice movement study IV—orientation of head lice with intact antennae and with distal antennae amputated.

	Lice with Amputated Antennae (*n* = 25)	Control Lice with Antennae (*n* = 25)
**Direction of Movement: Horizontal**		
proximal	7 (28%)	16 (64%)
distal	1 (4%)	-
no movement	17 (68%)	9 (36%)
**Direction of Movement: Vertical Hand-Highest**		
proximal	1 (4%)	3 (12%)
distal	8 (32%)	14 (56%)
no movement	16 (64%)	8 (32%)
**Direction of Movement: Vertical Hand-Lowest**		
proximal	5 (20%)	15 (60%)
distal	3 (12%)	-
no movement	17 (68%)	10 (40%)
